# Attractive combinations of female gingival displays, buccal corridor sizes, and facial heights according to orthodontists, dentists, and laypeople of different ages and sexes: a psychometric study

**DOI:** 10.1186/s13005-024-00417-1

**Published:** 2024-03-08

**Authors:** Ozra Niknam, Shakila Yousefi Hafshejani, Vahid Rakhshan

**Affiliations:** 1 Department of Orthodontics, School of Dentistry, Ahvaz Jundishapur University of Medical Sciences, Ahvaz, Iran; 2 School of Dentistry, Ahvaz Jundishapur University of Medical Sciences, Ahvaz, Iran; 3 Formerly, Department of Anatomy, Dental School, Azad University of Medical Sciences, Tehran, Iran

**Keywords:** Beauty, Esthetics, Dental Esthetics, Face, Sex dimorphism, Orthodontics, Dentistry, Maxillofacial surgery, Plastic surgery, Psychometrics, Art, Perception, Gingival display, Buccal corridor, Facial height

## Abstract

**Introduction:**

Esthetics plays a crucial role in orthodontics and many other dental and medical fields. To date, no study has assessed the combined effects of the 3 facial features ‘facial height, gingival display (GD), and buccal corridor size (BC)’ on facial/smile beauty. Therefore, this study was conducted for the first time.

**Methods:**

In this psychometric diagnostic study, beauty of 27 randomized perceptometric images of a female model with variations in facial heights (short, normal, long), gingival displays (0, 2, 4, 6 mm), and buccal corridor sizes (2%, 10%, 15%, 20%, 25%) were evaluated by 108 judges (36 orthodontists, 36 dentists, 36 laypeople) using a 5-scale Likert scale (1 to 5). Combined effects of facial heights, GDs, BCs, judges’ sexes, ages, and jobs, and their 2-way interactions were tested using a mixed-model multiple linear regression and a Bonferroni test. Zones of ideal features were determined for all judges and also for each group using repeated-measures ANOVAs and the Bonferroni test (α=0.05).

**Results:**

Judges’ sex but not their age or expertise might affect their perception of female beauty: men gave higher scores. The normal face was perceived as more beautiful than the long face (the short face being the least attractive). Zero GD was the most attractive followed by 4 mm; 6 mm was the least appealing. BCs of 15% followed by 10% were the most attractive ones, while 25% BC was the worst. The zone of ideal anatomy was: long face + 0mm GD + 15% BC; normal face + 2mm GD + 15% BC; long face + 2mm GD + 15% BC; normal face + 0mm GD + 15% BC.

**Conclusions:**

Normal faces, zero GDs, and 15% BCs may be the most appealing. Facial heights affect the perception of beauty towards GDs but not BCs.

**Supplementary Information:**

The online version contains supplementary material available at 10.1186/s13005-024-00417-1.

## Introduction

Beauty is a trendy topic as reflected by the number of papers published recently, or at any time, in reputable journals [1–6]. A beautiful appearance can have positive effects on self-confidence, mental health, social status, physical attractiveness, career success, academic success, intelligence, the level of happiness, or choice of spouse [1–5, 7].

Smile is a crucial factor to facial beauty [8]. It is the most important feature in facial attractiveness after the eyes [1–5, 8]. A warm smile is the universal language of kindness, and attracts affection and positive feedback and can even hide facial imperfections to some extent [1–5]. A beautiful smile is often the main complaint in dentistry, and patients usually evaluate the results of treatment based on positive changes in their smile [1, 3–5, 9]. Smile analysis includes the following factors: evaluation of the smile arch, gingival appearance, gingival beauty, examination of the buccal corridor space, and the fit between the dental and facial midlines [1, 2, 10, 11].

The perception of beauty is affected by many factors that can influence the subjective standards of observers, such as people’s culture, personal experiences, and the profession among others [1–5, 11]. Therefore, what is desirable from the point of view of dental aesthetics may be completely irrelevant to the patient’s point of view [1–5, 12]. Therefore, the classification of aesthetics into pleasant, acceptable, and unpleasant requires a calibration and a proper communication between the patient and the dentist [1, 3–5, 13].

Besides subjective factors related to the observer, anatomic factors of the observed face may matter as well. Dental and soft tissue feature affect esthetics in orthodontic treatment, although it is not clear which factors have the greatest impact on smile attractiveness at the end of the treatment [1–5, 12]. Many studies have been conducted to understand preferences of ordinary people and dentists toward the beauty factors of a smile [1–5].

The extent of gingival display is one of the factors that affect the beauty of a smile [1, 6, 9, 13, 14]. People who show too much gingiva in the upper jaw when they smile are called gummy smile [1, 6, 9, 13, 14]. This issue can decrease self-confidence and even cause psychological problems for people, making them seek treatment [15]. Although controversy exists over the normal level of gingival display, usually the display of maxillary incisors along with 1 to 2 mm of gingival margin is considered normal [16].

The width of the buccal corridor is another factor that may affect the attractiveness of a smile. A wide smile might be more attractive than a narrow one as far as it is not exaggerated [2, 8–10, 17–19]. Recently, some orthodontists refer to the buccal corridor as a negative space that should be limited by expanding the width of the maxilla. On the other hand, as it has been well shown in prosthetics, the lack of a buccal corridor space is one of the characteristics of artificially looking teeth [20].

An important point in smile esthetics is that most studies in this regard are limited to the mouth, neglecting the effect of the facial shape on the attractiveness of the smile [6]. The appearance of the face is an important factor in understanding the beauty of a smile. Therefore, the appearance of the face should be considered in the orthodontic treatment plan [17, 19]. When it comes to the combination of the 3 factors ‘facial forms with gingival displays and buccal corridor sizes’, there is no study in the literature.

Since there was no study on the effects of the combination of facial heights with gingival displays and buccal corridor sizes, we conducted this study. Its aim was to find the combined effects of gingival display and the width of the buccal corridor in each of the three facial shapes (long, normal, and short) on facial / smile esthetics from the perspectives of orthodontists versus general dentists verus laypeople. Moreover, the most appealing combinations of gingival displays and the widths of the buccal corridor and facial forms were comparatively determined for orthodontists, maxillofacial surgeons, and laypeople (and all of them combined). The null hypotheses were no effects of the abovementioned anatomical features as well as the judges' ages, sexes, and expertise on their esthetic preferences.

## Materials and methods

The survey was anonymous and did not collect any identifier or personal information of the judges apart from their anonymously taken age and sex and occupation; the results of the survey were completely confidential and used only for research purposes. The first page of the survey was an informed consent which asked the participants to begin the survey if they were consent to participate; yet the need for any signed informed consents by the survey participants were waived by the Institutional Review Board of Ahvaz Jundishapur University of Medical Sciences, Ahvaz, Iran (ethics approval code: IR.AJUMS.REC.1398.650). The photo-model signed informed consent allowing the researchers to use her image (either original or after photomanipulation) for the research and/or the article without the need for masking any parts of her face. The study protocol and its ethics were approved by the Institutional Review Board of Ahvaz Jundishapur University of Medical Sciences, Ahvaz, Iran (code: IR.AJUMS.REC.1398.650). All methods were performed in accordance with the relevant guidelines and regulations (including the Declaration of Helsinki); all experimental protocols were approved by the Institutional Review Board of Ahvaz Jundishapur University of Medical Sciences, Ahvaz, Iran.

### Sample size

The sample size of this study was calculated as 36 judges in each of the 3 groups to obtain powers above 90%, assuming an alpha of 0.05, and using the following parameters: 35.57±12.58 and 26.10±12.56 borrowed from a previous study on beauty factors [20]. The formula in use was:

$$N=\left[{\left({Z}_{1-\alpha }+{Z}_{1+\beta }\right)}^{2}\left({{\text{S}}}_{1}^{2}+{{\text{S}}}_{2}^{2}\right)\right]/{\left({X}_{1}-{X}_{2}\right)}^{2}$$

### Original photograph

A frontal smile photograph was taken from a young 23-year-old woman with a normal face with a class I occlusion, without crowding and spacing, with normal overjet and normal overbite, without any missing teeth, extracted teeth, or supernumerary teeth, without any dental prosthesis, without any implants, without lip asymmetry (a maximum difference of 25% between lips), with a normal gingival display of 1-2 mm in social smile mode, with normal size and proportions of the face in frontal view, and with a 15% buccal corridor width. The female model had to have no asymmetry or craniofacial syndrome, and no history of facial cosmetic procedures.

### Perceptometric image sets with controlled variable morphologies

Using the original photograph, 27 standardized images were created (using Adobe Photoshop, USA) representing different combinations of facial forms, gingival display extents, and buccal corridor widths:

A Facial forms

**Fig. 1:**
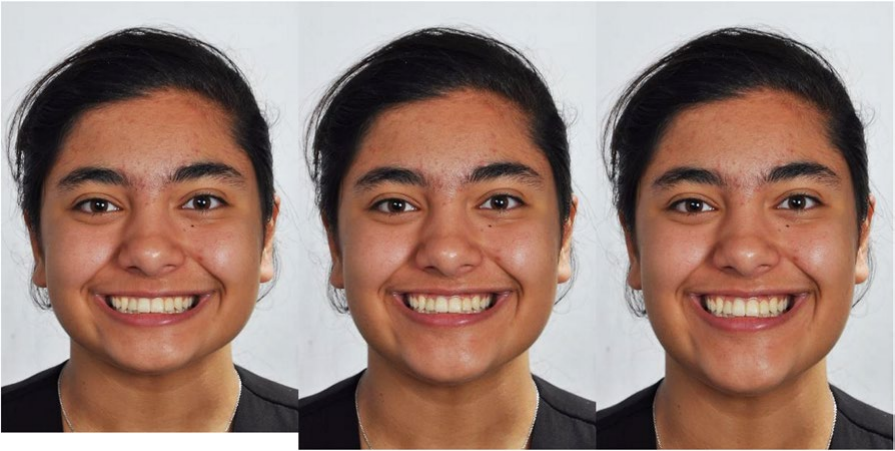
The first set of images for different facial shapes (left: short, middle: normal [the original image], right: long)

(1) Normal face from the original photograph, i.e., without change in the vertical height of the face (the ratio of the lower face to the middle height was equal to one). (2) Long face, in which the lower height of the face was increased by 10%. (3) Short face, in which the lower height of the face was reduced by 10% (Fig. 1). These 3 images were used to create a set of 12 images explained in subsection B and 15 images in subsection C below.

B Gingival display

**Fig. 2:**
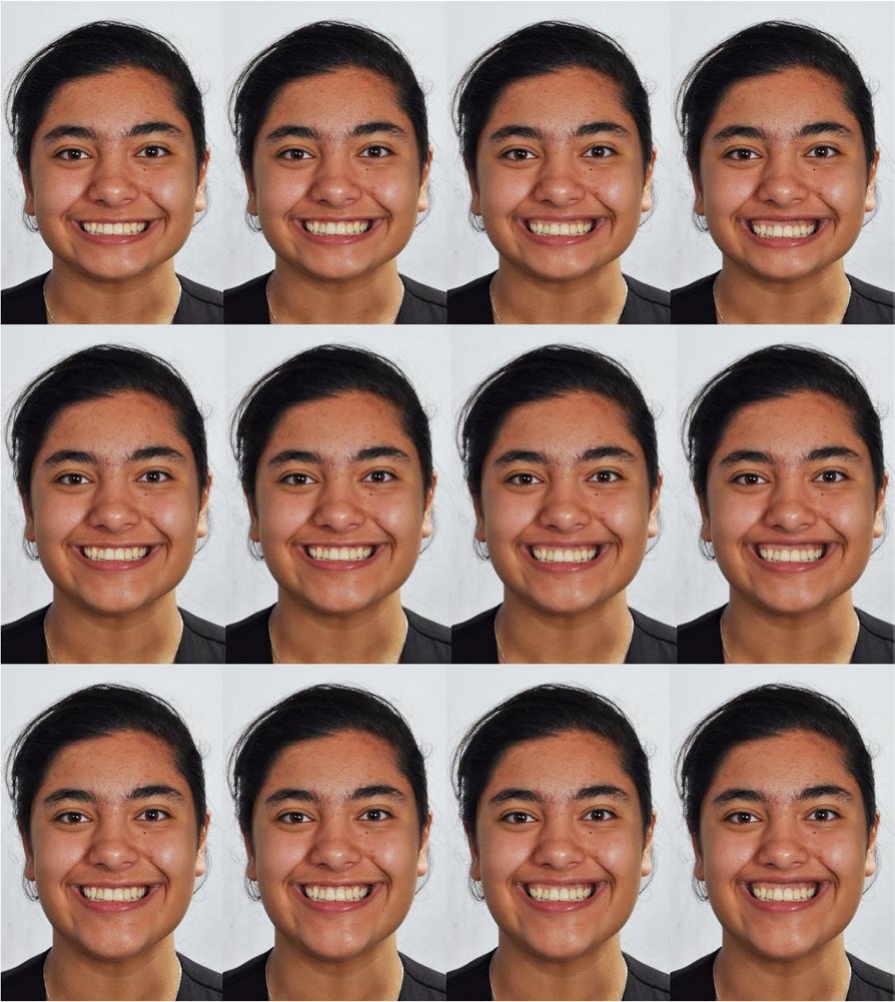
Various extents of gingival display, from left to right: 0 mm, 2 mm, 4 mm, and 6 mm. The top, middle, and bottom rows are respectively short, normal, and long faces

The amount of gingival display was defined as the distance from the zenith of the gingiva of the middle incisor to the lower border of the upper lip. Each of the above 3 images were used to create 4 new images (12 images in total, Fig. 2): By gradually altering the gingival display, for each of these three images with normal, long, and short facial patterns, 4 images were created using Photoshop, in which the gingival display was 6, 4, 2 [original], and 0 mm.

III. Buccal corridor width

**Fig. 3:**
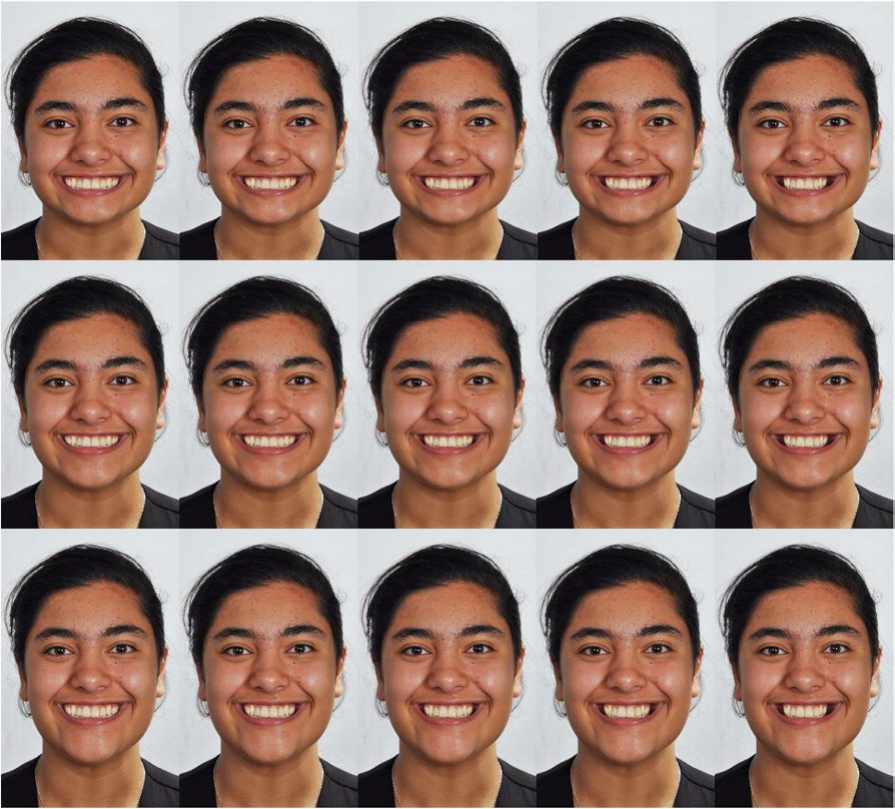
Various buccal corridor widths, from left to right: 2%, 10%, 15%, 20%, and 25%. The top, middle, and bottom rows are respectively short, normal, and long faces

The buccal corridor width was defined as the space between the buccal surface of the posterior teeth and the commissure of the lips when smiling. It was measured as the dark space between the labial commissure and the posterior teeth. calculated and measured. For creating these 15 images, the 3 images in subsection A above (‘Facial forms’) were used. The buccal corridor width on each of the three primary images (with a normal, long, short facial pattern with a normal gingival display of 2 mm) were photo-manipulated to create 5 images from each of the 3 images (amounting to 15 images). In each set of 5 images, the buccal corridor had these widths: 25%, 20%, 15% [original], 10%, and 2% (Fig. 3).

### Esthetic assessments

- Judges

Digital invitations were sent to various target groups until reaching the desired sample size. The minimum and maximum eligible ages for the respondents were 15 and 55 years, respectively. More than 250 judges rated some images of the survey, but many did so only partially. The sampling was continued until enrolling 108 judges (64 females, 44 males) who completed the full 27-image survey. These 108 raters were in 3 equal-size groups: 36 orthodontists, 36 general dentists, and 36 laypersons. The lay individuals had no knowledge of normal dentofacial proportions and angles; they were not educated and/or occupied in any field such as medicine, dentistry, painting, hairdressing, etc. These observers were selected randomly from various cultural and educational levels, so that their opinions could reflect the society's general understanding of beauty.

- Randomized Survey

The randomized digital survey was created by an orthodontist. It contained questions regarding each participant’s age, sex, education, and years of experience as well as 27 esthetic questions corresponding to the 27 perceptometric images that had been randomized. The respondents’ ages were recorded as 4 categories of between 15–24.9 (category A1), 25–34.9 (category A2), 35–44.9 (category A3), and 45–55 years old (category A4). Each judge was requested to rate the beauty of each of the images using a 5-point Numeric Rating Scale (NRS) with scores ranging from 1 to 5: 1 (very unattractive), 2 (unattractive), 3 (acceptable), 4 (attractive) to 5 (very attractive). The raters were instructed about the meaning of the beauty scores, i.e., that higher scores should be given to more appealing faces while lower scores indicate more unattractive faces. The judges were blinded to the original image. The beauty questions could be answered only once, that is once answered, the user could not scroll back to the previous image.

- Survey reliability

The internal consistency of the survey was excellent (Cronbach’s Alpha = 0.922, 95% CI = 0.900 to 0.942, *P* < 0.00000005).

### Statistical analysis

Descriptive statistics and 95% confidence intervals (CIs) were calculated for beauty scores of each of the 27 photographs in each of the 3 occupation groups. The sample normality was assessed and passed noting the central limit theorem and using histograms and q-q plots. The participants’ distributions in terms of age against job against sex were examined using a chi-squared test. The respondents’ sensitivity to the extent of changes in esthetic preferences as a function of photogrammetric stimuli (i.e., the Perceptometric serial anatomic alterations) was examined using a mixed-effects multiple linear regression followed by a Bonferroni post hoc test to examine the effects of each of the 3 dimensions of anatomic changes (i.e., facial forms, gingival displays, and buccal corridor widths) and their interactions on facial beauty; this analysis was also used to examine the effects of the raters’ age (4 levels [4 decades of life]), sex (2 levels [male, female]), and occupation (3 levels [control laypersons, general dentists, orthodontists]) on beauty scores they gave to the Perceptometric photographs showing anatomic modifications.

The ‘zone of ideal features’ was defined as the best anatomic combination of facial forms and midline statuses (i.e., the most beautiful image) as well as any other anatomic combinations (i.e., any other Perceptometric images) that were not significantly different from the best combination in terms of their esthetic scores. It was determined using a repeated-measures analysis of variance (RM-ANOVA) followed by a Bonferroni post hoc test. The software in use was SPSS 26 (IBM, Armonk, NY, USA). The level of significance was set at 0.05.

## Results

There were 46, 33, 22, and 7 participants in the age groups A1, A2, A3, and A4, respectively. Of the female participants, 35, 20, 8, and 1 were in the age groups A1, A2, A3, and A4, respectively. These numbers were 11, 13, 14, and 6 for males. According to the chi-squared test, these age/sex distributions were not similar (*P* = 0.001). In the groups ‘control, dentists, and orthodontists’ there were 26, 24, and 14 females, respectively. In the same groups, there were 10, 12, and 22 males, respectively. These sex/job distributions were not similar (chi-squared, *P* = 0.009). In the age categories A1, A2, A3, and A4, there were respectively 24, 7, 1, and 4 laypersons; 22, 13, 1, and 0 general dentists; and 0, 13, 20, and 3 orthodontists. The distributions of age against occupations were not even (chi-squared, *P* < 0.0005).

### Determinants of beauty

Descriptive statistics and 95% CIs for attractiveness scores given to each of the 27 photographs by male or female raters of each of the 3 occupations are presented in Table 1. The mixed-effects multiple regression’s characteristics were as follows: -2 Restricted Log Likelihood = 7624.774; Akaike's Information Criterion (AIC) = 7628.774; Hurvich and Tsai's Criterion (AICC) = 7628.778; Bozdogan's Criterion (CAIC) = 7642.665; Schwarz's Bayesian Criterion (BIC) = 7640.665.

**Table 1:** Descriptive statistics and 95% CIs for the beauty scores of each of the 27 perceptometric images according to laypersons, dentists, and orthodontists. In each image, the first number shows the extent of gingival display and the second number shows the buccal corridor width. Note: The perceptometric images are sorted in this table after data collection (for a better visualization); during the survey, the perceptometric images were randomized

		Laypeople							General Dentist							Orthodontist						
Image	Sex	N	Mean	SD	95% CI		M	Mx	N	Mean	SD	95% CI		M	Mx	N	Mean	SD	95% CI		M	Mx
Long 2mm 25%	Female	26	1.19	0.40	1.03	1.35	1	2	24	1.04	0.20	0.96	1.13	1	2	14	1.14	0.36	0.93	1.35	1	2
	Male	10	2.50	1.65	1.32	3.68	1	5	12	1.17	0.58	0.80	1.53	1	3	22	1.45	0.67	1.16	1.75	1	3
	Both	36	1.56	1.08	1.19	1.92	1	5	36	1.08	0.37	0.96	1.21	1	3	36	1.33	0.59	1.14	1.53	1	3
Short 2mm 25%	Female	26	1.35	0.56	1.12	1.57	1	3	24	1.04	0.20	0.96	1.13	1	2	14	1.29	0.61	0.93	1.64	1	3
	Male	10	2.10	1.10	1.31	2.89	1	4	12	1.17	0.58	0.80	1.53	1	3	22	1.73	0.70	1.42	2.04	1	3
	Both	36	1.56	0.81	1.28	1.83	1	4	36	1.08	0.37	0.96	1.21	1	3	36	1.56	0.69	1.32	1.79	1	3
Short 2mm 20%	Female	26	1.35	0.69	1.07	1.62	1	3	24	1.21	0.51	0.99	1.42	1	3	14	1.64	1.08	1.02	2.27	1	4
	Male	10	2.10	0.88	1.47	2.73	1	3	12	1.25	0.62	0.86	1.64	1	3	22	1.82	0.85	1.44	2.20	1	3
	Both	36	1.56	0.81	1.28	1.83	1	3	36	1.22	0.54	1.04	1.41	1	3	36	1.75	0.94	1.43	2.07	1	4
Long 2mm 20%	Female	26	1.54	0.86	1.19	1.89	1	4	24	1.33	0.64	1.06	1.60	1	3	14	1.43	0.85	0.94	1.92	1	4
	Male	10	2.30	1.42	1.29	3.31	1	5	12	1.75	0.87	1.20	2.30	1	4	22	1.91	0.75	1.58	2.24	1	4
	Both	36	1.75	1.08	1.38	2.12	1	5	36	1.47	0.74	1.22	1.72	1	4	36	1.72	0.81	1.45	2.00	1	4
Normal 2mm 25%	Female	26	1.62	0.85	1.27	1.96	1	4	24	1.58	1.10	1.12	2.05	1	4	14	1.36	0.74	0.93	1.79	1	3
	Male	10	2.50	0.97	1.80	3.20	1	4	12	1.67	0.98	1.04	2.29	1	4	22	1.73	0.88	1.34	2.12	1	4
	Both	36	1.86	0.96	1.54	2.19	1	4	36	1.61	1.05	1.26	1.97	1	4	36	1.58	0.84	1.30	1.87	1	4
Short 6mm 15%	Female	26	1.62	0.70	1.33	1.90	1	3	24	1.54	0.59	1.29	1.79	1	3	14	1.79	1.05	1.18	2.39	1	4
	Male	10	2.40	1.07	1.63	3.17	1	4	12	1.67	0.78	1.17	2.16	1	3	22	1.59	0.67	1.30	1.89	1	3
	Both	36	1.83	0.88	1.54	2.13	1	4	36	1.58	0.65	1.36	1.80	1	3	36	1.67	0.83	1.39	1.95	1	4
Long 2mm 15%	Female	26	1.85	0.97	1.46	2.24	1	4	24	1.46	0.72	1.15	1.76	1	3	14	2.29	0.99	1.71	2.86	1	4
	Male	10	2.70	0.95	2.02	3.38	1	4	12	1.67	0.65	1.25	2.08	1	3	22	2.36	0.79	2.01	2.71	1	4
	Both	36	2.08	1.02	1.74	2.43	1	4	36	1.53	0.70	1.29	1.76	1	3	36	2.33	0.86	2.04	2.62	1	4
Short 2mm 2%	Female	26	1.77	0.95	1.39	2.15	1	4	24	1.75	1.11	1.28	2.22	1	5	14	2.07	1.14	1.41	2.73	1	5
	Male	10	2.70	1.34	1.74	3.66	1	5	12	2.50	0.80	1.99	3.01	1	4	22	1.91	1.02	1.46	2.36	1	4
	Both	36	2.03	1.13	1.64	2.41	1	5	36	2.00	1.07	1.64	2.36	1	5	36	1.97	1.06	1.62	2.33	1	5
Normal 2mm 20%	Female	26	2.27	1.08	1.83	2.71	1	5	24	1.63	0.97	1.22	2.03	1	4	14	2.07	1.00	1.50	2.65	1	4
	Male	10	2.40	0.84	1.80	3.00	1	4	12	1.75	0.97	1.14	2.36	1	4	22	2.14	0.89	1.74	2.53	1	4
	Both	36	2.31	1.01	1.96	2.65	1	5	36	1.67	0.96	1.34	1.99	1	4	36	2.11	0.92	1.80	2.42	1	4
Long 6mm 15%	Female	26	2.31	1.19	1.83	2.79	1	5	24	1.88	0.68	1.59	2.16	1	3	14	1.93	1.07	1.31	2.55	1	4
	Male	10	2.20	0.92	1.54	2.86	1	4	12	1.92	0.79	1.41	2.42	1	3	22	2.14	0.77	1.79	2.48	1	3
	Both	36	2.28	1.11	1.90	2.65	1	5	36	1.89	0.71	1.65	2.13	1	3	36	2.06	0.89	1.75	2.36	1	4
Normal 6mm 15%	Female	26	2.35	1.26	1.84	2.86	1	5	24	2.00	0.83	1.65	2.35	1	3	14	1.86	0.86	1.36	2.36	1	3
	Male	10	2.50	0.85	1.89	3.11	1	4	12	2.08	1.08	1.39	2.77	1	4	22	2.00	0.62	1.73	2.27	1	3
	Both	36	2.39	1.15	2.00	2.78	1	5	36	2.03	0.91	1.72	2.34	1	4	36	1.94	0.71	1.70	2.19	1	3
Long 2mm 2%	Female	26	1.85	0.78	1.53	2.16	1	3	24	1.88	0.99	1.46	2.29	1	4	14	2.57	1.28	1.83	3.31	1	5
	Male	10	2.20	1.40	1.20	3.20	1	4	12	2.33	1.44	1.42	3.25	1	5	22	2.36	1.14	1.86	2.87	1	4
	Both	36	1.94	0.98	1.61	2.28	1	4	36	2.03	1.16	1.64	2.42	1	5	36	2.44	1.18	2.04	2.84	1	5
Short 2mm 15%	Female	26	1.85	0.88	1.49	2.20	1	4	24	2.00	0.83	1.65	2.35	1	4	14	2.71	1.07	2.10	3.33	1	5
	Male	10	2.60	1.07	1.83	3.37	1	4	12	1.92	0.67	1.49	2.34	1	3	22	2.41	1.10	1.92	2.90	1	4
	Both	36	2.06	0.98	1.72	2.39	1	4	36	1.97	0.77	1.71	2.23	1	4	36	2.53	1.08	2.16	2.89	1	5
Short 2mm 10%	Female	26	1.69	0.84	1.35	2.03	1	3	24	1.88	0.90	1.50	2.25	1	4	14	2.29	0.99	1.71	2.86	1	4
	Male	10	3.10	0.88	2.47	3.73	2	4	12	2.92	1.16	2.18	3.66	1	5	22	2.45	1.30	1.88	3.03	1	5
	Both	36	2.08	1.05	1.73	2.44	1	4	36	2.22	1.10	1.85	2.59	1	5	36	2.39	1.18	1.99	2.79	1	5
Long 2mm 10%	Female	26	2.08	1.13	1.62	2.53	1	5	24	2.17	1.01	1.74	2.59	1	4	14	2.79	1.05	2.18	3.39	1	4
	Male	10	2.30	0.95	1.62	2.98	1	4	12	2.75	1.06	2.08	3.42	1	4	22	2.73	0.88	2.34	3.12	1	5
	Both	36	2.14	1.07	1.78	2.50	1	5	36	2.36	1.05	2.01	2.72	1	4	36	2.75	0.94	2.43	3.07	1	5
Short 4mm 15%	Female	26	2.15	0.78	1.84	2.47	1	5	24	2.46	0.83	2.11	2.81	1	4	14	2.57	1.34	1.80	3.35	1	5
	Male	10	2.80	1.32	1.86	3.74	1	5	12	2.50	1.09	1.81	3.19	1	4	22	2.82	0.66	2.52	3.11	2	4
	Both	36	2.33	0.99	2.00	2.67	1	5	36	2.47	0.91	2.16	2.78	1	4	36	2.72	0.97	2.39	3.05	1	5
Normal 2mm 15%	Female	26	2.31	1.16	1.84	2.78	1	5	24	2.33	1.09	1.87	2.79	1	4	14	2.93	1.14	2.27	3.59	1	5
	Male	10	3.10	1.10	2.31	3.89	1	5	12	2.08	0.90	1.51	2.66	1	4	22	2.68	0.99	2.24	3.12	1	5
	Both	36	2.53	1.18	2.13	2.93	1	5	36	2.25	1.02	1.90	2.60	1	4	36	2.78	1.05	2.42	3.13	1	5
Normal 2mm 2%	Female	26	2.35	1.16	1.88	2.82	1	5	24	2.08	1.35	1.51	2.65	1	5	14	3.00	1.41	2.18	3.82	1	5
	Male	10	3.40	0.97	2.71	4.09	2	5	12	2.67	0.98	2.04	3.29	1	4	22	2.55	1.26	1.99	3.11	1	5
	Both	36	2.64	1.20	2.23	3.04	1	5	36	2.28	1.26	1.85	2.70	1	5	36	2.72	1.32	2.27	3.17	1	5
Normal 2mm 10%	Female	26	2.38	1.10	1.94	2.83	1	5	24	2.54	1.06	2.09	2.99	1	5	14	3.14	1.29	2.40	3.89	1	5
	Male	10	3.00	0.82	2.42	3.58	1	4	12	2.42	0.79	1.91	2.92	1	4	22	2.77	1.11	2.28	3.26	1	5
	Both	36	2.56	1.05	2.20	2.91	1	5	36	2.50	0.97	2.17	2.83	1	5	36	2.92	1.18	2.52	3.32	1	5
Short 0mm 15%	Female	26	2.27	1.15	1.80	2.73	1	5	24	3.04	0.91	2.66	3.43	2	5	14	2.79	1.37	2.00	3.58	1	5
	Male	10	3.00	1.33	2.05	3.95	1	5	12	2.67	1.37	1.80	3.54	1	4	22	2.77	1.07	2.30	3.25	1	5
	Both	36	2.47	1.23	2.06	2.89	1	5	36	2.92	1.08	2.55	3.28	1	5	36	2.78	1.17	2.38	3.17	1	5
Normal 4mm 15%	Female	26	2.85	1.19	2.37	3.33	1	5	24	2.42	1.02	1.99	2.85	1	5	14	2.50	1.02	1.91	3.09	1	5
	Male	10	3.10	0.57	2.69	3.51	2	4	12	3.08	0.51	2.76	3.41	2	4	22	2.77	0.75	2.44	3.11	2	4
	Both	36	2.92	1.05	2.56	3.27	1	5	36	2.64	0.93	2.32	2.95	1	5	36	2.67	0.86	2.38	2.96	1	5
Long 4mm 15%	Female	26	2.69	1.26	2.18	3.20	1	5	24	2.67	0.96	2.26	3.07	1	5	14	2.57	1.09	1.94	3.20	1	5
	Male	10	3.50	0.71	2.99	4.01	3	5	12	2.58	0.79	2.08	3.09	1	4	22	3.00	0.82	2.64	3.36	2	4
	Both	36	2.92	1.18	2.52	3.32	1	5	36	2.64	0.90	2.33	2.94	1	5	36	2.83	0.94	2.51	3.15	1	5
Short 2mm 15%	Female	26	2.54	1.14	2.08	3.00	1	4	24	2.75	0.99	2.33	3.17	1	5	14	3.36	1.28	2.62	4.09	1	5
	Male	10	3.10	0.88	2.47	3.73	2	4	12	2.25	1.06	1.58	2.92	1	4	22	3.09	0.81	2.73	3.45	1	4
	Both	36	2.69	1.09	2.33	3.06	1	4	36	2.58	1.02	2.24	2.93	1	5	36	3.19	1.01	2.85	3.54	1	5
Normal 0mm 15%	Female	26	3.12	1.31	2.59	3.64	1	5	24	2.92	1.02	2.49	3.35	1	5	14	3.14	1.29	2.40	3.89	1	5
	Male	10	2.90	0.74	2.37	3.43	2	4	12	2.67	1.30	1.84	3.49	1	4	22	3.27	0.83	2.91	3.64	2	5
	Both	36	3.06	1.17	2.66	3.45	1	5	36	2.83	1.11	2.46	3.21	1	5	36	3.22	1.02	2.88	3.57	1	5
Long 2mm 15%	Female	26	3.12	1.28	2.60	3.63	1	5	24	2.67	0.92	2.28	3.05	1	4	14	3.50	1.09	2.87	4.13	2	5
	Male	10	3.30	0.48	2.95	3.65	3	4	12	2.50	0.67	2.07	2.93	2	4	22	3.41	0.80	3.06	3.76	2	5
	Both	36	3.17	1.11	2.79	3.54	1	5	36	2.61	0.84	2.33	2.89	1	4	36	3.44	0.91	3.14	3.75	2	5
Normal 2mm 15%	Female	26	3.23	1.07	2.80	3.66	1	5	24	3.17	0.92	2.78	3.55	2	5	14	3.36	1.08	2.73	3.98	1	5
	Male	10	3.10	0.99	2.39	3.81	2	5	12	3.00	0.60	2.62	3.38	2	4	22	3.50	0.67	3.20	3.80	2	5
	Both	36	3.19	1.04	2.84	3.55	1	5	36	3.11	0.82	2.83	3.39	2	5	36	3.44	0.84	3.16	3.73	1	5
Long 0mm 15%	Female	26	3.50	0.99	3.10	3.90	2	5	24	3.42	1.06	2.97	3.86	2	5	14	3.64	1.08	3.02	4.27	2	5
	Male	10	3.10	0.74	2.57	3.63	2	4	12	3.25	0.87	2.70	3.80	2	4	22	3.45	0.74	3.13	3.78	2	5
	Both	36	3.39	0.93	3.07	3.71	2	5	36	3.36	0.99	3.03	3.70	2	5	36	3.53	0.88	3.23	3.82	2	5

*SD* Standard deviation, *M* Minimum, *Mx* Maximum; *CI* Confidence interval

**Fig. 4:**
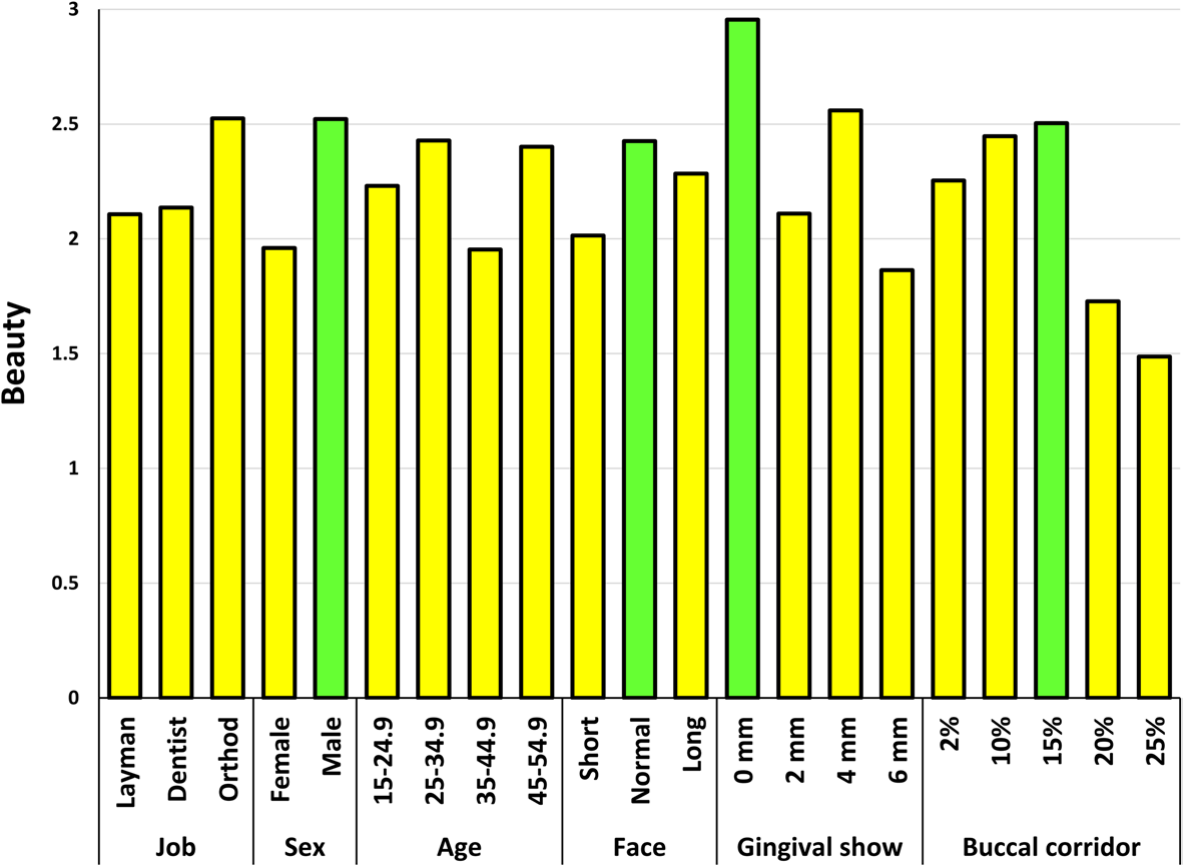
Estimated marginal means for esthetic scores within the expertise, sex, and age of the judges and different facial features of the photo model. The green bars highlight significantly more attractive features according to the mixed-model multiple regression and Bonferroni post hoc test

The mixed-effects multiple linear regression identified the following factors as significant (Fig. 4, Tables 2 and 3): the judge’s sex and the photomodel’s facial height, gingival display, and buccal corridor (Fig. 4, Tables 2 and 3). The judge’s sex significantly interacted with the photomodel’s gingival display and buccal corridor in terms of beauty scores (Table 3). Similarly, the judge’s occupation significantly interacted with the photomodel’s gingival display and buccal corridor in terms of beauty scores (Table 3). Additionally, the judge’s age interacted with the photomodel’s gingival display (Table 3). The photomodel’s facial height interacted with the extent of her gingival display (Table 3).

**Table 2:** Estimated marginal means within the mixed-model regression framework

Aspect	Variable	Level	Mean	SE	95% CI	
Judge	Expertise	Laypeople	2.107	0.214	1.681	2.533
		General Dentist	2.136	0.221	1.697	2.576
		Orthodontist	2.525	0.131	2.265	2.784
	Sex	Female	1.96	0.182	1.600	2.321
		Male	2.522	0.127	2.269	2.775
	Age	15-24.9	2.231	0.109	2.014	2.448
		25-34.9	2.428	0.105	2.220	2.637
		35-44.9	1.954	0.269	1.419	2.490
		45-54.9	2.401	0.328	1.749	3.053
Photo model	Face	Short	2.014	0.113	1.790	2.238
		Normal	2.426	0.113	2.202	2.650
		Long	2.284	0.113	2.059	2.508
	Gingival show	0 mm	2.955	0.123	2.711	3.199
		2 mm	2.11	0.110	1.891	2.329
		4 mm	2.56	0.123	2.316	2.804
		6 mm	1.864	0.123	1.620	2.108
	Buccal corridor	2%	2.254	0.123	2.010	2.498
		10%	2.447	0.123	2.203	2.691
		15%	2.504	0.111	2.283	2.724
		20%	1.728	0.123	1.484	1.972
		25%	1.487	0.123	1.243	1.731

*SE*, Standard error, *CI* Confidence interval.

**Table 3:** The results of the mixed-effects multiple linear regression analysis

Predictor	F	*P*
Intercept	385.783	0.000
Judge’s Sex	8.792	0.004
Job	0.453	0.637
Age	1.217	0.306
Facial Height	14.258	0.000001
Gingival Display	64.003	0.000000
Buccal Corridor	82.381	0.000000
Sex × Job	1.979	0.144
Sex × Age	0.789	0.503
Sex × Facial Height	1.726	0.178
Sex × Gingival Display	5.060	0.002
Sex × Buccal Corridor	3.801	0.004
Job × Age	1.298	0.277
Job × Facial Height	2.087	0.080
Job × Gingival Display	2.680	0.014
Job × Buccal Corridor	3.011	0.002
Age × Facial Height	1.433	0.198
Age × Gingival Display	2.030	0.033
Age × Buccal Corridor	0.821	0.628
Facial Height × Gingival Display	6.279	0.000001
Facial Height × Buccal Corridor	0.710	0.683

According to the Bonferroni test, the normal facial height, a 0-mm gingival display (followed by a 4-mm gingival show), and a 15% buccal corridor width (followed by a 10% width) were the most attractive features (Table 4, Fig. 4). The least attractive features were the short face, 6 mm of gingival show, and a 25% buccal corridor width (Table 4, Fig. 4).

**Table 4:** The results of the Bonferroni post hoc test following the mixed-model regression

Variable	Item I	Item J	Difference (I-J)	SE	*P*	95% CI	
Facial height	Normal	Short	0.412	0.051	0.000	0.290	0.534
	Long	Short	0.269	0.051	0.000	0.147	0.392
		Normal	-0.142	0.051	0.016	-0.265	-0.020
Gingival show	2 mm	0 mm	-0.845	0.067	0.000	-1.021	-0.668
	4 mm	0 mm	-0.394	0.087	0.000	-0.624	-0.165
		2 mm	0.450	0.067	0.000	0.274	0.626
	6 mm	0 mm	-1.090	0.087	0.000	-1.320	-0.861
		2 mm	-0.246	0.067	0.001	-0.422	-0.070
		4 mm	-0.696	0.087	0.000	-0.926	-0.466
Buccal corridor width	10%	2%	0.193	0.087	0.265	-0.051	0.437
	15%	2%	0.250	0.068	0.002	0.059	0.441
		10%	0.057	0.068	1.000	-0.134	0.248
	20%	2%	-0.525	0.087	0.000	-0.769	-0.281
		10%	-0.718	0.087	0.000	-0.963	-0.474
		15%	-0.775	0.068	0.000	-0.966	-0.584
	25%	2%	-0.766	0.087	0.000	-1.011	-0.522
		10%	-0.960	0.087	0.000	-1.204	-0.715
		15%	-1.016	0.068	0.000	-1.207	-0.826
		20%	-0.241	0.087	0.056	-0.486	0.003

*SE* Standard error, *CI* Confidence interval

### Zone of ideal features

**Fig. 5.**
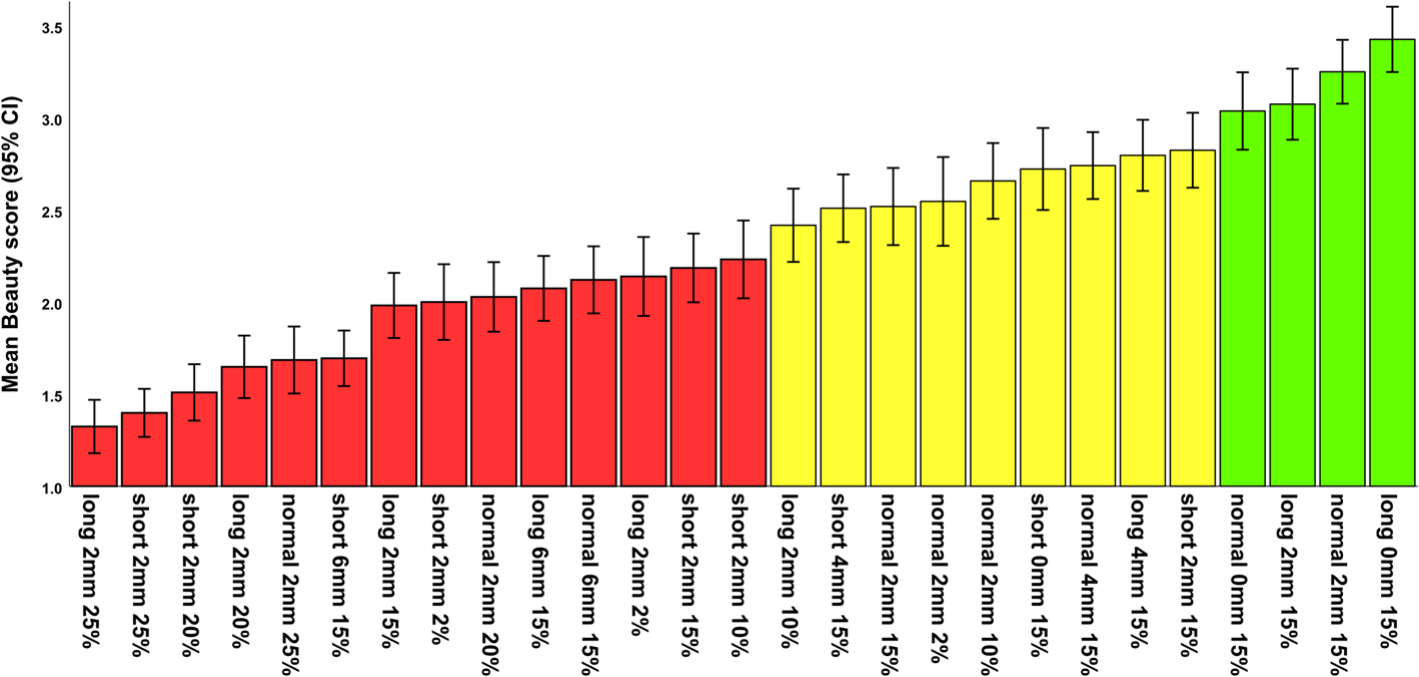
Estimated marginal means and 95% CIs for beauty scores pertaining to each image (n for each bar = 108). The green bars show the zone of ideal anatomy. Note: The perceptometric images are sorted after data collection (for a better visualization); during the survey, the perceptometric images were randomized

**Fig. 6:**
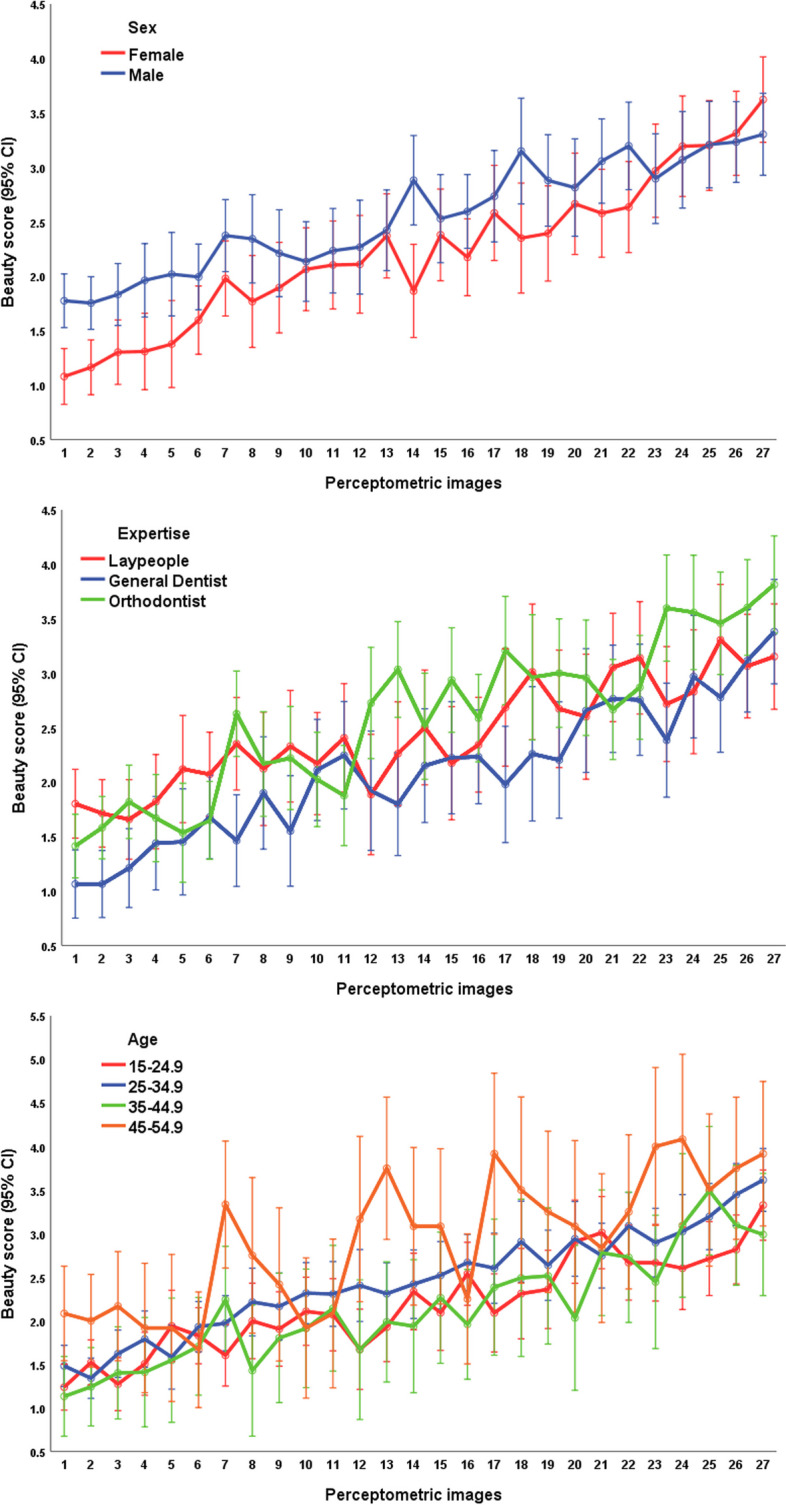
Estimated marginal means and 95% CIs for beauty scores pertaining to each image. Perceptometric images 1 to 27 respectively denote the following combinations of “facial form, gingival display, and buccal corridor width”: long 2mm 25%, short 2mm 25%, short 2mm 20%, long 2mm 20%, normal 2mm 25%, short 6mm 15%, long 2mm 15%, short 2mm 2%, normal 2mm 20%, long 6mm 15%, normal 6mm 15%, long 2mm 2%, short 2mm 15%, short 2mm 10%, long 2mm 10%, short 4mm 15%, normal 2mm 15%, normal 2mm 2%, normal 2mm 10%, short 0mm 15%, normal 4mm 15%, long 4mm 15%, short 2mm 15%, normal 0mm 15%, long 2mm 15%, normal 2mm 15%, long 0mm 15%. Note: The perceptometric images are sorted in these images after data collection (for a better visualization); during the survey, the perceptometric images were randomized

According the repeated-measures ANOVA and the post hoc Bonferroni test, the most beautiful image was the long face with 0 mm of gingival display and a 15% buccal corridor width, while the zone of ideal anatomy (*P* > 0.05, Bonferroni, Figs. 5 and 6, Tables 5 and 6, Appendix 1) were the abovementioned image as well as these 3 images: a normal face, with 0 mm gingival show and 15% buccal corridor, a long face with 2 mm gingival display and 15% buccal corridor, and a normal face with a 2mm gingival display and 15% buccal corridor. The rest of images had esthetic scores significantly smaller than the most beautiful image (*P* < 0.05, Bonferroni, Figs. 5 and 6, Tables 5 and 6, Appendix 1).

**Table 5:** Estimated marginal means for esthetic scores of the 27 perceptometric images. N for each image is 108 judges. The first number shows the extent of gingival display and the second number shows the buccal corridor width. Note: The perceptometric images are sorted in this table after data collection (for a better visualization); during the survey, the perceptometric images were randomized

Image	Mean	SE	95% CI	
Long 2mm 25%	1.448	0.089	1.270	1.626
Short 2mm 25%	1.476	0.088	1.302	1.650
Short 2mm 20%	1.583	0.103	1.378	1.788
Long 2mm 20%	1.655	0.122	1.412	1.899
Normal 2mm 25%	1.717	0.139	1.440	1.993
Short 6mm 15%	1.807	0.109	1.590	2.024
Long 2mm 15%	2.188	0.120	1.949	2.427
Short 2mm 2%	2.073	0.147	1.780	2.365
Normal 2mm 20%	2.063	0.145	1.775	2.351
Long 6mm 15%	2.102	0.133	1.838	2.365
Normal 6mm 15%	2.173	0.141	1.893	2.452
Long 2mm 2%	2.193	0.156	1.882	2.504
Short 2mm 15%	2.398	0.135	2.130	2.665
Short 2mm 10%	2.403	0.149	2.107	2.699
Long 2mm 10%	2.458	0.147	2.166	2.750
Short 4mm 15%	2.397	0.123	2.152	2.641
Normal 2mm 15%	2.662	0.152	2.360	2.965
Normal 2mm 2%	2.774	0.176	2.424	3.124
Normal 2mm 10%	2.651	0.153	2.348	2.954
Short 0mm 15%	2.744	0.162	2.421	3.066
Normal 4mm 15%	2.832	0.141	2.553	3.112
Long 4mm 15%	2.933	0.146	2.643	3.222
Short 2mm 15%	2.930	0.149	2.634	3.227
Normal 0mm 15%	3.128	0.161	2.808	3.447
Long 2mm 15%	3.205	0.144	2.919	3.491
Normal 2mm 15%	3.270	0.135	3.003	3.537
Long 0mm 15%	3.453	0.137	3.182	3.725

*SE* Standard error, *CI* Confidence interval

**Table 6:** The results of the Bonferroni test comparing the most and least attractive images with all other images. The full results of the Bonferroni test are presented as Appendix 1. The first number shows the extent of gingival display and the second number shows the buccal corridor width. Note: The perceptometric images are sorted in this table after data collection (for a better visualization); during the survey, the perceptometric images were randomized

Image I	Image J	Mean Difference (I-J)	SE	*P*	95% CI	
Long 2mm 25% (the least attractive image)	Short 2mm 25%	-0.074	0.063	1.0	-0.321	0.173
	Short 2mm 20%	-0.185	0.075	1.0	-0.480	0.109
	Long 2mm 20%	-0.324	0.067	0.001497	-0.588	-0.060
	Normal 2mm 25%	-0.361	0.090	0.037124	-0.715	-0.007
	Short 6mm 15%	-0.370	0.087	0.015245	-0.713	-0.028
	Long 2mm 15%	-0.657	0.078	0.000000	-0.965	-0.350
	Short 2mm 2%	-0.676	0.094	0.000000	-1.046	-0.306
	Normal 2mm 20%	-0.704	0.095	0.000000	-1.079	-0.328
	Long 6mm 15%	-0.750	0.100	0.000000	-1.146	-0.354
	Normal 6mm 15%	-0.796	0.103	0.000000	-1.204	-0.388
	Long 2mm 2%	-0.815	0.103	0.000000	-1.221	-0.409
	Short 2mm 15%	-0.861	0.093	0.000000	-1.230	-0.492
	Short 2mm 10%	-0.907	0.104	0.000000	-1.318	-0.497
	Long 2mm 10%	-1.093	0.107	0.000000	-1.516	-0.669
	Short 4mm 15%	-1.185	0.093	0.000000	-1.553	-0.818
	Normal 2mm 15%	-1.194	0.097	0.000000	-1.578	-0.811
	Normal 2mm 2%	-1.222	0.119	0.000000	-1.690	-0.754
	Normal 2mm 10%	-1.333	0.108	0.000000	-1.758	-0.908
	Short 0mm 15%	-1.398	0.115	0.000000	-1.853	-0.943
	Normal 4mm 15%	-1.417	0.102	0.000000	-1.819	-1.014
	Long 4mm 15%	-1.472	0.100	0.000000	-1.866	-1.079
	Short 2mm 15%	-1.500	0.110	0.000000	-1.933	-1.067
	Normal 0mm 15%	-1.713	0.118	0.000000	-2.180	-1.246
	Long 2mm 15%	-1.750	0.114	0.000000	-2.200	-1.300
	Normal 2mm 15%	-1.926	0.102	0.000000	-2.327	-1.525
	Long 0mm 15%	-2.102	0.109	0.000000	-2.533	-1.671
Long 0mm 15% (the most attractive image)	Long 2mm 25%	2.102	0.109	0.000000	1.671	2.533
	Short 2mm 25%	2.028	0.105	0.000000	1.614	2.441
	Short 2mm 20%	1.917	0.112	0.000000	1.473	2.361
	Long 2mm 20%	1.778	0.116	0.000000	1.318	2.237
	Normal 2mm 25%	1.741	0.122	0.000000	1.258	2.223
	Short 6mm 15%	1.731	0.116	0.000000	1.274	2.189
	Long 2mm 15%	1.444	0.107	0.000000	1.022	1.867
	Short 2mm 2%	1.426	0.129	0.000000	0.917	1.935
	Normal 2mm 20%	1.398	0.111	0.000000	0.961	1.835
	Long 6mm 15%	1.352	0.100	0.000000	0.955	1.748
	Normal 6mm 15%	1.306	0.115	0.000000	0.852	1.759
	Long 2mm 2%	1.287	0.118	0.000000	0.823	1.751
	Short 2mm 15%	1.241	0.111	0.000000	0.804	1.678
	Short 2mm 10%	1.194	0.136	0.000000	0.659	1.730
	Long 2mm 10%	1.009	0.118	0.000000	0.544	1.475
	Short 4mm 15%	0.917	0.112	0.000000	0.473	1.361
	Normal 2mm 15%	0.907	0.117	0.000000	0.444	1.370
	Normal 2mm 2%	0.880	0.152	0.000026	0.279	1.480
	Normal 2mm 10%	0.769	0.131	0.000016	0.253	1.284
	Short 0mm 15%	0.704	0.121	0.000021	0.227	1.180
	Normal 4mm 15%	0.685	0.111	0.000004	0.249	1.122
	Long 4mm 15%	0.630	0.097	0.000001	0.246	1.013
	Short 2mm 15%	0.602	0.123	0.001314	0.115	1.089
	Normal 0mm 15%	0.389	0.103	0.097614	-0.019	0.797
	Long 2mm 15%	0.352	0.098	0.172146	-0.034	0.738
	Normal 2mm 15%	0.176	0.103	1.0	-0.229	0.581

*SE* Standard error, *CI* Confidence interval

**Fig. 7:**
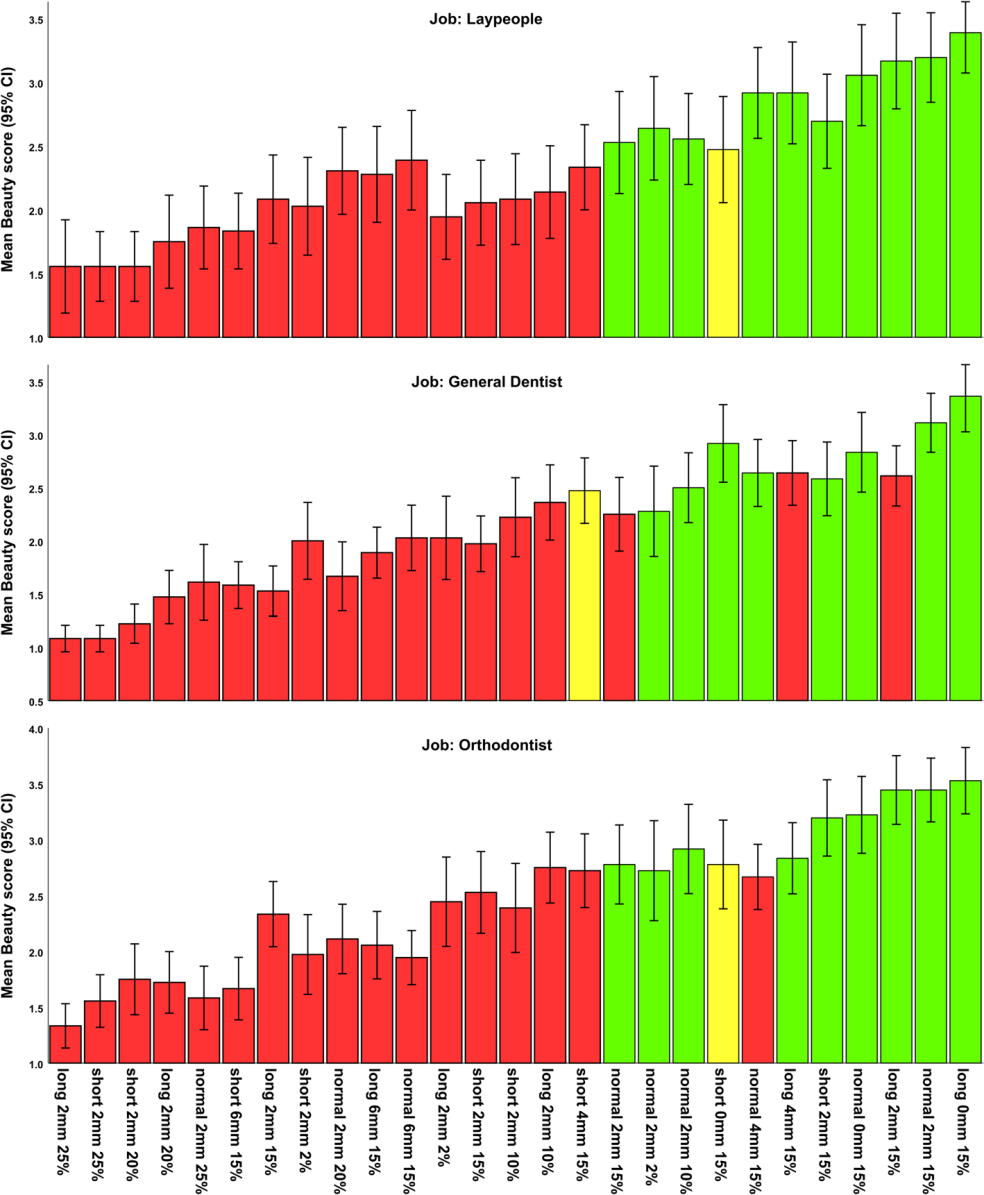
Estimated marginal means and 95% CIs for beauty scores pertaining to each image by each group (n for each bar = 36). The green bars show the images with statistically insignificant differences at *P* > 0.1. The yellow bars show images with marginally significant differences. The order of the bars is the same for all 3 job groups. Note: The perceptometric images are sorted in these images after data collection (for a better visualization); during the survey, the perceptometric images were randomized.

The zones of ideal esthetics were also estimated for each of the 3 groups of judges (Fig. 7, Table 7, Appendix 2). All groups identified the same image as the most attractive one (long face, no gingival display, 15% buccal corridor). Each group’s preferences for about 9 or 10 other images were not significantly different from this top image (Fig. 7, Table 7, Appendix 2).

**Table 7:** The zones of ideal anatomy from the perspectives of laypeople, dentists, and orthodontists (*P* > 0.05, Bonferroni). The full results of the Bonferroni test are presented as Appendix 2. The first number shows the extent of gingival display and the second number shows the buccal corridor width. Note: The perceptometric images are sorted in this table after data collection (for a better visualization); during the survey, the perceptometric images were randomized

Expertise	Image (I)	Image (J)	Mean Difference (I-J)	SE	*P*	95% CI	
Laypeople	Long 0mm 15% (the most attractive image)	Long 2mm 25%	1.833	0.224	0.000000	0.879	2.788
		Short 2mm 25%	1.833	0.189	0.000000	1.027	2.640
		Short 2mm 20%	1.833	0.193	0.000000	1.009	2.658
		Long 2mm 20%	1.639	0.226	0.000006	0.674	2.603
		Normal 2mm 25%	1.528	0.213	0.000008	0.620	2.436
		Short 6mm 15%	1.556	0.216	0.000008	0.633	2.478
		Long 2mm 15%	1.306	0.218	0.000270	0.377	2.234
		Short 2mm 2%	1.361	0.246	0.001126	0.311	2.412
		Normal 2mm 20%	1.083	0.180	0.000252	0.316	1.851
		Long 6mm 15%	1.111	0.177	0.000119	0.355	1.867
		Normal 6mm 15%	1.000	0.191	0.002776	0.184	1.816
		Long 2mm 2%	1.444	0.197	0.000005	0.604	2.285
		Short 2mm 15%	1.333	0.218	0.000194	0.402	2.265
		Short 2mm 10%	1.306	0.238	0.001333	0.288	2.323
		Long 2mm 10%	1.250	0.208	0.000274	0.360	2.140
		Short 4mm 15%	1.056	0.178	0.000333	0.296	1.815
		Normal 2mm 15%	0.861	0.219	0.131781	-0.073	1.795
		Normal 2mm 2%	0.750	0.277	1.000000	-0.433	1.933
		Normal 2mm 10%	0.833	0.227	0.282101	-0.136	1.803
		Short 0mm 15%	0.917	0.223	0.080174	-0.036	1.869
		Normal 4mm 15%	0.472	0.201	1.0	-0.386	1.331
		Long 4mm 15%	0.472	0.189	1.0	-0.334	1.279
		Short 2mm 15%	0.694	0.202	0.548345	-0.170	1.559
		Normal 0mm 15%	0.333	0.203	1.0	-0.534	1.201
		Long 2mm 15%	0.222	0.187	1.0	-0.578	1.022
		Normal 2mm 15%	0.194	0.168	1.0	-0.524	0.912
Dentist	Long 0mm 15% (the most attractive image)	Long 2mm 25%	2.278	0.181	0.000000	1.506	3.050
		Short 2mm 25%	2.278	0.181	0.000000	1.506	3.050
		Short 2mm 20%	2.139	0.192	0.000000	1.321	2.957
		Long 2mm 20%	1.889	0.210	0.000000	0.993	2.785
		Normal 2mm 25%	1.750	0.234	0.000003	0.753	2.747
		Short 6mm 15%	1.778	0.200	0.000000	0.926	2.630
		Long 2mm 15%	1.833	0.180	0.000000	1.063	2.603
		Short 2mm 2%	1.361	0.236	0.000558	0.353	2.369
		Normal 2mm 20%	1.694	0.214	0.000001	0.781	2.607
		Long 6mm 15%	1.472	0.171	0.000000	0.741	2.203
		Normal 6mm 15%	1.333	0.222	0.000262	0.386	2.280
		Long 2mm 2%	1.333	0.215	0.000142	0.417	2.249
		Short 2mm 15%	1.389	0.184	0.000003	0.604	2.173
		Short 2mm 10%	1.139	0.262	0.039183	0.022	2.256
		Long 2mm 10%	1.000	0.236	0.054009	-0.006	2.006
		Short 4mm 15%	0.889	0.217	0.084746	-0.039	1.817
		Normal 2mm 15%	1.111	0.221	0.005190	0.168	2.055
		Normal 2mm 2%	1.083	0.291	0.242961	-0.159	2.326
		Normal 2mm 10%	0.861	0.252	0.578359	-0.217	1.939
		Short 0mm 15%	0.444	0.216	1.0	-0.478	1.367
		Normal 4mm 15%	0.722	0.189	0.187947	-0.087	1.531
		Long 4mm 15%	0.722	0.141	0.004104	0.118	1.326
		Short 2mm 15%	0.778	0.259	1.0	-0.326	1.881
		Normal 0mm 15%	0.528	0.201	1.0	-0.331	1.386
		Long 2mm 15%	0.750	0.146	0.003606	0.128	1.372
		Normal 2mm 15%	0.250	0.205	1.0	-0.623	1.123
Orthodontist	Long 0mm 15% (the most attractive image)	Long 2mm 25%	2.194	0.153	0.000000	1.540	2.849
		Short 2mm 25%	1.972	0.171	0.000000	1.241	2.703
		Short 2mm 20%	1.778	0.200	0.000000	0.926	2.630
		Long 2mm 20%	1.806	0.168	0.000000	1.088	2.524
		Normal 2mm 25%	1.944	0.187	0.000000	1.148	2.741
		Short 6mm 15%	1.861	0.188	0.000000	1.061	2.662
		Long 2mm 15%	1.194	0.137	0.000000	0.610	1.779
		Short 2mm 2%	1.556	0.189	0.000000	0.750	2.361
		Normal 2mm 20%	1.417	0.171	0.000000	0.688	2.146
		Long 6mm 15%	1.472	0.171	0.000000	0.741	2.203
		Normal 6mm 15%	1.583	0.175	0.000000	0.835	2.332
		Long 2mm 2%	1.083	0.201	0.001686	0.227	1.940
		Short 2mm 15%	1.000	0.169	0.000350	0.278	1.722
		Short 2mm 10%	1.139	0.208	0.001291	0.253	2.025
		Long 2mm 10%	0.778	0.160	0.008495	0.095	1.460
		Short 4mm 15%	0.806	0.190	0.055616	-0.007	1.618
		Normal 2mm 15%	0.750	0.166	0.024051	0.041	1.459
		Normal 2mm 2%	0.806	0.221	0.304268	-0.139	1.750
		Normal 2mm 10%	0.611	0.200	1.000000	-0.244	1.466
		Short 0mm 15%	0.750	0.184	0.089002	-0.036	1.536
		Normal 4mm 15%	0.861	0.183	0.013913	0.079	1.643
		Long 4mm 15%	0.694	0.173	0.104034	-0.043	1.432
		Short 2mm 15%	0.333	0.169	1.0	-0.388	1.055
		Normal 0mm 15%	0.306	0.125	1.0	-0.227	0.839
		Long 2mm 15%	0.083	0.156	1.0	-0.584	0.750
		Normal 2mm 15%	0.083	0.161	1.0	-0.605	0.772

*SE* standard error, *CI* Confidence interval

## Discussion

Effects of gingival display and buccal corridor with smile esthetic has been assessed in previous research [1, 2, 6, 8–10, 14, 17–22]. Most of the studies on smile beauty limited their assessments to the mouth [20], but few have investigated the effect of face shape on smile attractiveness [6]. The facial shape may play an important role in the beauty of smile; changes in smile appearance are perceived differently depending on vertical facial differences [17, 19]. For this reason, we examined three long, normal and short facial forms. It has been suggested by some authors that age or sex of the observer might not influence their esthetic perception [20, 22–24], while the photomodel’s sex might influence the judges’ perception in some cases [25]. Still, since most studies in this regard have assessed exclusively female smiles (like the present study) [24, 26–29], the latter suggestion needs more research [20]. The present study was not in agreement with the former observation, as we noted differences between esthetic perceptions of male and female judges.

Gingival display was found to be the most attractive at zero followed by 4 mm. From the perspective of all three groups of general dentists, orthodontists and ordinary people, and in all three faces, increased gingival displays for 4 or 6 mm were unattractive and needed treatment in order to create an ideal smile. De Lima et al [6] investigated the effect of facial form on smile attractiveness with different gingival displays from the perspective of dentists and laypeople. They observed a statistically significant difference between different levels of gingival show for both normal and tall faces. In their study [6], normal people were less sensitive than dentists and considered a smaller range for people with normal to long face patterns. In the aforementioned study, the normal face was better for all levels of gingival display in the eyes of both ordinary people and experts, which is also the case in our study. Rajeev et al [21] investigated the role of different buccal corridor widths 2%, 10%, 15%, 22%, 28% in perception of smile beauty between general dentists and lay people. They found no significant difference in judgment of general dentists and laypeople; in general, both preferred smiles with a narrow or medium buccal corridor width. In our study, participants preferred smiles with medium and narrow buccal corridor widths compared to wide ones. Oz et al [20] investigated the differences in perception of smile attractiveness with different amounts of gingival display and buccal corridor width between four groups (orthodontists, prosthodontists, oral and maxillofacial surgeons, normal people). They concluded that 0 and 12% of buccal corridor widths were the most beautiful [20]. The same authors [20] asserted that the most attractive smiles had either +2 or −3 mm gingival displays [20].

This study was limited by some factors. Like most previous studies on smile esthetics, we as well only evaluated female smiles. The reason was that the inclusion of 27 additional images for male smiles would make the questionnaire excessively long and deterring many respondents. The addition of more photomodels instead of photo-manipulating the same model could improve the generalizability of results. However, it would no more be considered a Perceptometric approach, because the latter needs all facial features except the ones being studied to be reserved constant. Future studies should take into account the addition of more photomodels from both sexes and even from various age and ethnic groups. With increasing age of the model, the ratings of attractiveness for the criteria ‘female gingival displays, buccal corridor sizes, and facial heights’ may be different, possibly also depending on the age of the referee. All such interactions seem intriguing and deserve to be researched in the future. However, such generous additions of photomodels may need compensations in other departments, for instance by reducing the number of Perceptometric images per photomodel. This is because if the Perceptometric method is to be used, as per each added photomodel, a similar number of new Perceptometric images need to be added to the questionnaire, making it excessively large and thus difficult to complete. Hence, studies should find a balance between the number of variables in question and the difficulty of the resulted questionnaire. Another interesting idea is to evaluate whether models with Angle Classes I, II or III may be evaluated differently by dental experts (e.g., orthodontists, dentists, prosthodontists) versus laypeople. All these interesting ideas warrant future research. As another limitation, although the group sizes were equal, they were not balanced in terms of distributions of sexes, expertise, or age; nevertheless, the used statistical analyses compounded with the very large size of the observations (around 3000 datapoints) were able to account for any such imbalances. Future studies should also evaluate additional anatomical features. They should as well examine more groups of judges. The generalizability of our results may be limited to the culture and ethnic background of this population. Also, its generalizability is limited to female facial anatomy.

## Conclusions

Further studies should be conducted as an outlook in which the above suggestions for improvement can be implemented. The present paper should therefore be regarded as a pilot study. Within the limitations of this study, it could be concluded that judges’ sex but not their age or expertise might affect their perception of female smile / facial beauty: men tended to give higher scores. The normal face was perceived as more beautiful than the long face (the short face was the least attractive). Zero gingival displays followed by 4 mm were the most attractive ones and those at 6 mm were the least appealing ones. Buccal corridors with sizes of 15% followed by 10% were the most attractive ones, while a 25% buccal corridor was the worst.

The combinations of these 3 facial features made some images the most attractive ones: the long face with 0 mm of gingival display and 15% of buccal corridor width was the most beautiful image, followed by the normal face with 2 mm of gingival display and 15% buccal corridor, followed by the long face with 2 mm of exposed gingiva and 15% buccal corridor, and finally the normal face with 0 mm gingival show and 15% buccal corridor.

Judges’ sexes interact with their perception of female gingival exposures and buccal corridor sizes, but not female facial heights. Although esthetic scores of different jobs were not different, still referees’ jobs could affect their sensitivities to esthetic perception of both gingival displays and buccal corridors. Similarly, their age interacted with the perception of gingival exposure esthetics. Female facial heights may affect the perception of beauty of referees towards the extents of female gingival display but not their preferences of female buccal corridor sizes.

### Supplementary Information

**Additional file 1: Appendix 1.** The results of the Bonferroni post hoc test after the repeated-measures ANOVA (*n* = 108 judges × 27 images); this table shows pairwise differences between each two perceptometric images.

**Additional file 2: Appendix 2.** The results of the Bonferroni post hoc test after the repeated-measures ANOVA for each group (*n* = 36 judges per group × 27 images); this table shows pairwise differences between each two perceptometric images.

## Data Availability

The data are available from the corresponding author upon request.
